# Characterization of Two Loss-of-Function NF1 Variants in Chinese Patients and Potential Molecular Interpretations of Phenotypes

**DOI:** 10.3389/fgene.2021.660592

**Published:** 2021-05-11

**Authors:** Tingting Zhang, Tianting Han, Zhiya Dong, Chuanyin Li, Wenli Lu

**Affiliations:** ^1^Department of Pediatrics, Ruijin Hospital Affiliated to Shanghai Jiao Tong University, Shanghai, China; ^2^State Key Laboratory of Molecular Biology, Shanghai Institute of Biochemistry and Cell Biology, Center for Excellence in Molecular Cell Science, Chinese Academy of Sciences, Shanghai, China; ^3^Cancer Center, Shanghai Tenth People’s Hospital, School of Medicine, Tongji University, Shanghai, China

**Keywords:** neurofibromatosis type 1, *NF1* mutation, Ras/ErK, ubiquitination, hypertension, short stature

## Abstract

Neurofibromatosis type 1 (NF1) is a common genetic disorder characterized by cafe’-au-lait spots, skinfold freckles, the formation of neurofibromas, skeletal dysplasia, vascular dysplasia, and an increased risk of malignant tumors. In this study, two Chinese NF1 children troubled with bone lesions or hypertension were reported. A *de novo NF1* mutation (c.4925T > A/p.V1642E) and a maternally inherited *NF1* mutation (c.4883T > A/p.L1628^∗^) were identified by molecular sequence. According to the ACMG/AMP guidelines, the c.4925T > A was classified as variants of uncertain significance (VOUS) while the c.4883T > A mutation was identified as likely Pathogenic. Further study found that these two NF1 mutants had lost their function to inhibit the Ras/Erk signaling and the proliferation of cells, which could interpretate some phenotypes of these two NF1 patients. We also observed these two NF1 mutants displayed decreased protein stability with increased ubiquitination levels compared with that of wild-type NF1.

## Introduction

Neurofibromatosis type 1 (NF1; OMIM#162200) is an autosomal-dominant inherited genetic disorder with an updated incidence of approximately 1 in 2,500 (Corsello et al., 2018). NF1 predisposes affected individuals to multisystem problems, ranging from pigmentary abnormalities (Café-au-lait macules/CALMs, freckling and Lisch nodules) and long-bone dysplasia (pseudoarthrosis, focal or widespread decreased bone mineral density and short stature) to tumor predisposition syndrome (optic pathway glioma, cutaneous neurofibroma, and plexiform neurofibroma) and vascular anomalies (hypertension due to renal artery stenosis or cardiac valvular defects, cerebrovascular disease) (Yao et al., 2019). The diagnostic criteria for NF1 were originally released by the National Institutes of Health (NIH) Consensus Development Conference in 1987. It is specified that NF1 individuals satisfy at least two of these clinical features included in the NIH Criteria (No author list, 1988b; Gutmann et al., 1997).

Neurofibromatosis type 1 results from mutations in the *NF1* gene mapping on human chromosome 17 (Barker et al., 1987) that encoding neurofibromin protein (DeClue et al., 1991; Gutmann et al., 1991). It is well established that a small central domain of neurofibromin protein sequence resembles the GTPase activating family of proteins (GAP) (Martin et al., 1990), a region therefore termed the GAP-related domain (GRD), and plays negative control on Ras signaling pathways: PI3 kinase/Akt/mammalian target of rapamycin (mTOR) (Harrisingh and Lloyd, 2004; Johannessen et al., 2005)and Raf/MEK/ERK (Wang et al., 2012). Since neurofibromin works as a tumor suppressor protein, impaired neurofibromin leads to upregulated Ras activity and increased cell growth, which is consistent with the tumor predisposition syndrome and aggregation cells in vessels observed in NF1 patients (Sasaki et al., 1995; Ly and Blakeley, 2019). Short stature is estimated to be observed in 13–33% of individuals with NF1 (Szudek et al., 2000; Vassilopoulou-Sellin et al., 2000) whereas the molecular and cellular etiology for growth deficit in affected patients has not been elucidated. Several *Nf1*-deficient murine models showing similar bony abnormalities with NF1 patients had been reported (Kolanczyk et al., 2007; Schindeler et al., 2008; Wang et al., 2010), and investigators suggested that NF1 is a major regulator of development and growth of the skeleton. Sharma et al. (2013) demonstrated that hyperactive Ras/MAPK is a potential pathway underlying the pathogenesis of NF1-associated bone formation and remodeling deficits. Here we report two symptomatic children bearing with heterozygous *NF1* mutations, and we are positioned to determine whether the specific NF1 mutants from our patients are loss-of-function ones that can recapitulate the experimental trends of above *Nf1*- deficient animal models.

## Materials and Methods

### Editorial Policies and Ethical

Two Chinese patients with *NF1* mutants that never reported previously were recruited. This study was approved by the Institutional Review Board of the Ruijin Hospital. The informed consent was obtained from each participant considerations.

### Molecular Investigations

DNA was extracted from peripheral blood leukocytes using DNA extraction kit (Qiagen, Hilden, Germany). Mutations were identified by next generation sequencing (NGS) on custom gene panel for genetic neuroblastoma which contains *NF1* gene. The result was confirmed by Sanger sequencing and validated by parental testing. We followed the ACMG/AMP guidelines for variant pathogenicity assessment (Nykamp et al., 2017).

### Plasmid Construction

The full-length cDNA of *NF1* was synthesized by GENEWIZ (Suzhou, China) and inserted into pCDNA3.0 plasmid with N-terminus flag tagged. Mutations of *NF1* (V1642E and L1628^∗^) was introduced by site-directed mutagenesis as previously reported (Li et al., 2020b). The sgRNA targeting *NF1* gene was designed using an online tool^1^ as previously reported (Xu et al., 2018). The designed sgRNA were synthesized as oligos (Sangon, Shanghai, China), annealed and inserted into a PX330 vector that was digested with *Bbs*I.

### Cell Culture and Transfection

The human neuroblastoma cell line SHSY5Y was purchased from the American Type Culture Collection (ATCC, Manassas, VA, United States) and was cultured in DMEM supplemented with 10% fetal bovine serum (FBS, Gibco, Carlsbad, CA, United States) and penicillin/streptomycin (Gibco) in a 37°C humidified atmosphere of 5% CO2. Plasmids were transfected into SHSY5Y cells using Lipofectamine^®^ 2000 (Thermo Fisher, Waltham, MA, United States) according to the manufacturer’s instructions.

### Reverse Transcription-Quantitative PCR

Total RNA was extracted from cells using a total RNA kit (Tiangen, Beijing, China). Complementary DNA (cDNA) was synthesized using ReverTra Ace qPCR RT Master Mix (Toyobo, Osaka, Japan). The RT-qPCR assay was performed using the SYBR Green Master Mix (Toyobo, Osaka, Japan) with the CFX96 real-time PCR system (Bio-Rad Laboratories, CA, United States), according to the manufacturer’s protocol. The relative abundances of *NF1* gene were normalized to that of *GAPDH*, using the 2^ΔΔCt^ method (Li et al., 2020a). The specific sequences of primers used in this study are listed in Table 1. All the data were obtained from three independent experiments and data were analyzed using one-way ANOVA with Tukey’s *post hoc* test.

**TABLE 1:** Sequences of the primers used in RT-Qpcr.

Target gene	Forward primer (5′-3′)	Reverse primer (5′-3′)
*GAPDH*	GAGTCAACGGATT TGGTCGTATTG	ATTTGCCATGGGTGG AATCATATTG
*NF1*	CAAGTAAGCCATTCT CAAGAGGCAG	GGAGTGAATTTA CCAGCACATAGTGA

### Immunoprecipitation and Immunoblotting

For immunoprecipitation, cells were lysed in RIPA buffer [50 mM Tris–HCl, 150 mM NaCl, 5 mM EDTA, 0.1% sodium dodecyl sulfate (SDS), and 1% NP-40] supplemented with a protease inhibitor cocktail. Then, cell lysates were incubated with anti-Flag affinity gels (Merck, Kenilworth, NJ, United States) overnight at 4°C. The immunoprecipitates were enriched and denatured at 100°C for 10 min in 2 × SDS-PAGE loading buffer. The inputs, immunoprecipitates and other cell lysates were then subjected to SDS-PAGE and transferred to a PVDF membrane (Bio-Rad, United States). The membranes were incubated with the appropriate antibodies against GAPDH (1:5000, 60004-1-Ig, Proteintech, China), NF1 (1:500, 27249-1-AP, Proteintech), Flag (1:1000, 20543-1-AP, Proteintech), pan-Ras (1:2,000, MABS195, Millipore, Germany), Erk1/2 (1:3,000, SAB1305560, Millipore) or phospho-Erk1/2 (1:500, E7028, Millipore). Secondary antibodies were labeled with HRP, and the signals were visualized using Tanon 5200 Imaging System (Tanon, China).

### Ras-GTP Assay

Ras-GTP levels were detected as previously described (Sharma et al., 2013). Briefly, SHSY5Y cells were lysed in non-ionic lysis buffer (20 mM Tris-Cl PH7.6, 137 mM NaCl, 1 mM EGTA, 1% Triton-X-100, 10% glycerol, 1.5 mM MgCl_2_) supplemented with protease inhibitor cocktails, and the Ras activity was determined using a Ras activation assay kit (Millipore). Briefly, GTP-bound Ras levels were determined by incubating cell lysates with Raf-1 Ras-binding domain conjugated to agarose beads followed by an immunoblot using an anti-pan-Ras antibody (Millipore) and the total Ras was also detected.

### NF1^–/–^ Knockout Cell Line

A *NF1*^–^*^/^*^–^ knockout cell line was generated using the CRISPR-CAS9 technique as previously described (Li et al., 2020b). Briefly, SHSY5Y cells were transfected with CRISPR-CAS9-based sgRNA (PX330-NF1-sgRNA), and monoclonal were chosen and detected by immunoblotting analysis. Then, genetic ablation of *NF1* was confirmed by first generation sequencing.

### Cell Proliferation Assay

Three thousand cells were seeded into a 96-well plate. The 0 h time point was defined as 6 h after the cells were seeded. After 24, 48, and 72 h, the cells were incubated with Cell Counting Kit-8 (CCK8) solution (Beyotime, Jiangsu, China) for 2 h at 37°C. Then, the product was quantified spectrophotometrically at a wavelength of 450 nm using a microplate reader (Bio-Rad, CA, United States). The experiments were conducted with six replicates and repeated three times.

### Statistical Analysis

All experiments were performed in triplicate. All values are depicted as the mean ± standard deviation (SD). One-way ANOVA was performed with Tukey’s *post hoc* multiple comparisons test using GraphPad Prism 7.0 (GraphPad Software, United States). A *P*-value of <0.05 was considered statistically significant, while a *P*-value of <0.01 was considered very significant.

## Results

### Identification of *NF1* Mutation

**FIGURE 1:**
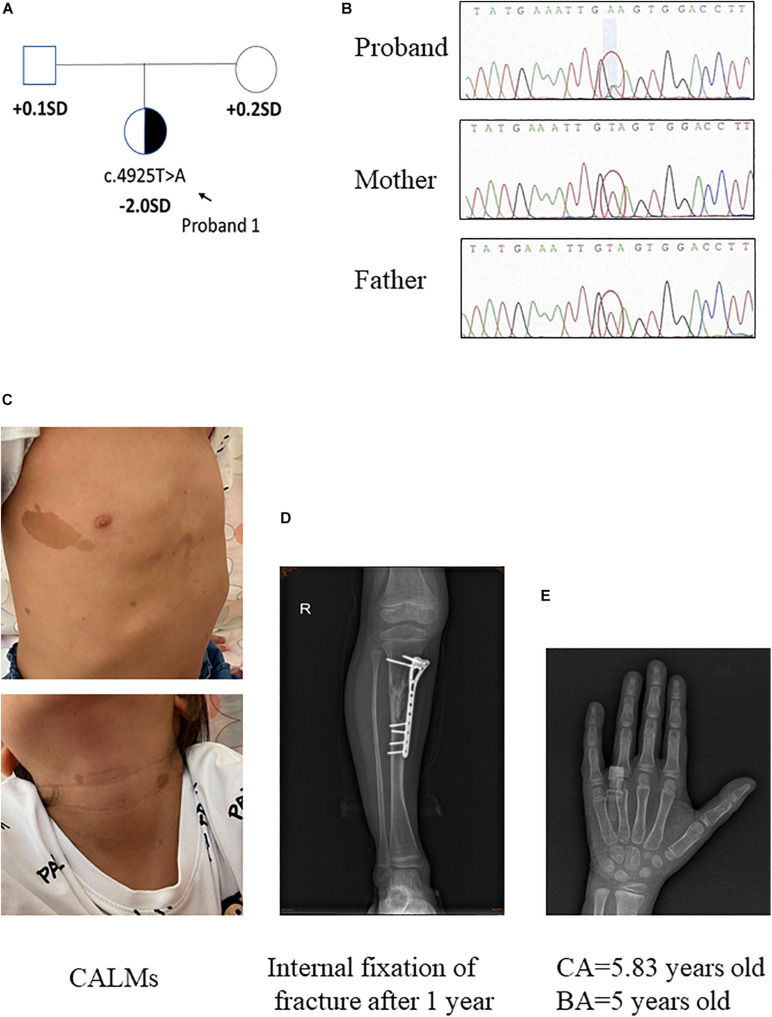
Clinical information of patient 1. **(A,B)** Pedigree of the family with a *de novo* c.4925T > A mutation in *NF1* gene, and the partial sequencing chromatograph of the family members. **(C)** CALMs of patient 1. **(D)** Bone fracture: X-ray examination on right tibia showed internal fixation of fracture 1 year later. **(E)** X-ray examination of proband 1 at the age of 5.83 years old showing a delayed bone age (5 years old) (by the Greulich–Pyle method).

**FIGURE 2:**
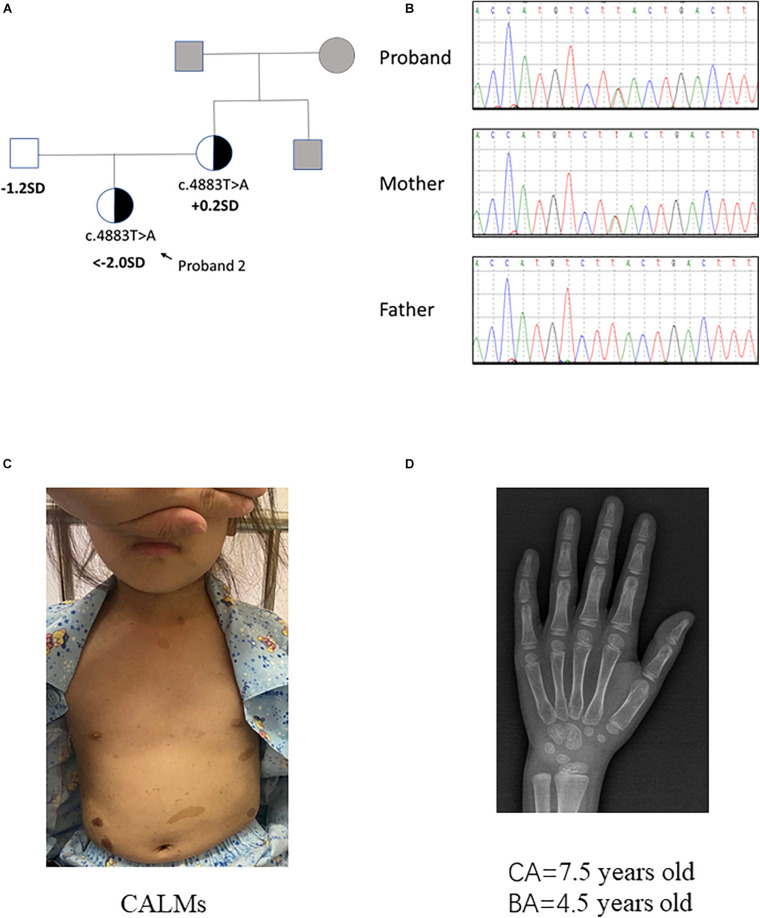
Clinical information of patient 2. **(A,B)** Pedigree of the family with a maternally inherited c.4883T > A mutation in *NF1* gene, and the partial sequencing chromatograph of the family members. Squares and circles in gray indicate suspectable family members who had not received mutation analysis. **(C)** CALMs of patient 2. **(D)** X-ray examination of proband 2 at the age of 7.5 years old showing a delayed bone age (4.5 years old) (by the Greulich–Pyle method).

Pedigrees of the family with *NF1* mutation and results of mutation analysis by direct DNA sequencing were shown in Figures 1A,B, 2A,B, respectively. Proband 1 was identified a c.4925T > A (p.V1642E, NM_001042492.2) mutation in *NF1* gene that was *de novo*. Proband 2 was identified a c.4883T > A (p.L1628^∗^, NM_001042492) mutation, which was maternally inherited. According to the ACMG/AMP guidelines, the c.4925T > A mutation was identified as variants of uncertain significance (VOUS) while the c.4883T > A mutation was identified as likely Pathogenic.

### Clinical Characteristics

The proband 1 was vaginally delivered at 37 weeks of gestation after an uneventful pregnancy. Her birth weight and birth length were 3.5 kg (75th percentile) and 50 cm (50th percentile), respectively. This patient reached developmental milestones at appropriate age. The child was born with CALMs which were scattered over her whole body (number > 6, with the longest axis > 4 cm) (Figure 1C). At 4 years old, she had a sudden fracture on her right tibia during a gentle physical activity and had an internal fixation of fracture (Figure 1D). Later the histopathological examination of the bone lesion revealed a non-ossifying fibroma. Another NF1-associated characteristic of this child was short stature (height: 108.7 cm, −2 SD). Routine laboratory test findings (e.g., blood routine, urine routine, liver function, urine function, blood sugar, blood lipids, electrolytes, etc.) were within normal limits. Arginine GH test showed that the patient had a mild growth hormone deficiency for a peak GH level as 9.057 ng/ml. Insulin-like growth factor 1 level was within the low level of reference ranges (72 ng/ml, normal range: 50–286 ng/ml). The thyroid function was normal. Magnetic resonance imaging on brain had no positive findings. When the patient was 5 years and 10 months old/5.83 years old, bone age x-ray was almost delayed by 1 year (by the Greulich–Pyle method, Figure 1E). Eye examination had no finding of Lisch nodules. Her parents were healthy, within average height, and there was no history of NF1-associated features. Collectively, patient 1 presented CALMs, axillary freckles, short stature (growth hormone deficiency), bone lesion with non-ossifying fibroma. She had no intracalvarium neurofibromas and cutaneous neurofibromas, no visible Lisch nodules, no positive family history.

Proband 2 was delivered at 36 weeks of gestation by cesarean section due to an unknown reason. Her birth weight and birth length were 2.5 kg (3rd percentile) and 50 cm (50th percentile), respectively. This patient reached developmental milestones at appropriate age. When the patient was 7 years and 5 months old, she came to our hospital for recurrent high blood pressure (maximal value of 160/100 mmHg) and paroxysmal palpitation. Metoprolol can relieve her discomfort. The clinical examination showed CALMs scattered on her trunk and axillary freckles (number > 6, with the longest axis > 3 cm) (Figure 2C). In addition, the height of this patient was obviously short (114.0 cm, < −2 SD). Routine laboratory test findings (e.g., blood routine, urine routine, liver function, urine function, blood sugar, blood lipids, electrolytes, etc.) were within normal limits. Stimulation testing demonstrated an incomplete growth hormone deficiency with the peak GH levels of 5.987 ng/ml and thyroid function tests was normal. Insulin-like growth factor 1 level was 156 ng/ml (normal range: 50–286 ng/ml). Plasma metanephrine value was normal (38.3 pg/ml, normal range: 14–90 pg/ml). Serum ACTH, aldosterone and renin activity were within reference ranges. The early morning (8:00 am) and 24 h urinary-free cortisol levels in this patient is normal. Bone age x-ray revealed a delayed bone age of 4.5 years old when the patient was 7 years and 6 months old/7.5 years old (by the Greulich–Pyle method, Figure 2D). Examination on multiple organs including echocardiography, ultrasound on abdominal cavity/post-peritoneum/arteria renalis, chest radiography, computed tomographic (CT) angiography of the abdominal vessels and enhanced CT of adrenal gland all showed negative finding. X-ray on four limbs showed no abnormity. Dynamic electrocardiogram found atrial premature beats. Since the patient had no complaint of eye discomfort, she refused the eye examination. The patient’s mother presented classic NF1 symptoms like typical CALMs and axillary freckles. Multiple subcutaneous nodules appeared during the mother’s puberty and some noticeable nodules were removed by dermatologist. It is said the mother’s brother had similar NF1 symptoms but the detailed information was not available. In brief, patient 2 had positive phenotypes including CALMs, axillary freckles, short stature (growth hormone deficiency), unexplained hypertension and similar family history. Conversely, she had no cutaneous neurofibromas, no Lisch nodules, no complaint hinting at optic glioma, no distinctive osseous lesion, etc.

### Mutations in the *NF1* Gene Cause Decreased Protein Levels *in vitro*

**FIGURE 3:**
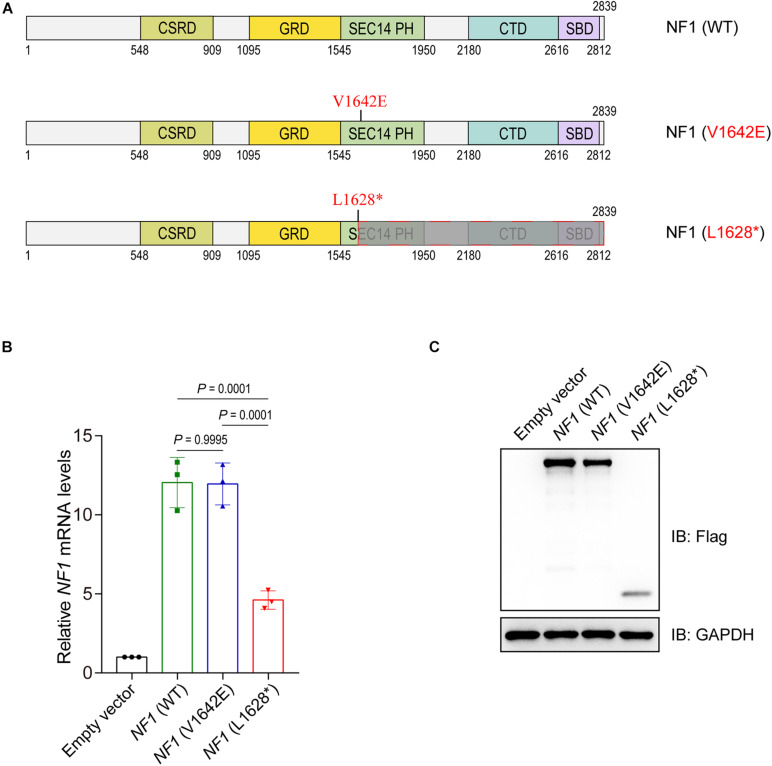
Mutations in the *NF1* gene cause decreased protein levels. **(A)** Schematic view of human wild type or mutant NF1 proteins involved in this study. CSRD, Cysteine-Serine-rich domain; GRD, GTPase-activating protein-related domain; SEC14-PH, SEC14 domain and pleckstrin homology (PH) domain; CTD, Carboxy-terminal domain; SBD, Syndecan-binding domain. WT, wild type. **(B)** Detect the mRNA levels of wild type or mutant NF1 by quantitative PCR (qPCR). Empty vector, wild type NF1, or mutant NF1 (V1642E and L1628*) were transfected into SHSY5Y cells. Total RNAs were extracted, complementary DNA (cDNA) was synthesized, and then the mRNA levels of *NF1* and *GAPDH* were detected by qPCR. The data are expressed as mean ± SD and were analyzed using one-way ANOVA with Tukey’s *post hoc* test, three independent experiments. **(C)** Detect the protein levels of wild type NF1 or mutant NF1 by immunoblotting analysis. Empty vector, or Flag tagged wild type NF1 or mutant NF1 (V1642E and L1628*) were transfected into SHSY5Y cells, and the indicated protein levels were detected by immunoblotting analysis.

Two mutations of *NF1* are involved in this study, one expressed a single amino acid substituted NF1 (V1642E) protein and the other expressed truncated NF1 (L1628^∗^) protein (Figure 3A). The eukaryotic expression plasmids of wild type *NF1* and mutant *NF1* (V1642E and L1628^∗^) were constructed with N-terminal Flag tag. The mRNA levels of *NF1* (L1628^∗^) is much lower than that of wild type and *NF1* (V1642E) (Figure 3B), this phenomenon might be attributed to non-sense-mediated mRNA decay (NMD) which is a translation-coupled mechanism that eliminates mRNAs containing premature translation-termination codons (PTCs). However, both the protein levels of mutant NF1 (V1642E and L1628^∗^) is less than that of wild type one (Figure 3C).

### Mutations of *NF1* Cause Decreased Protein Stability and Increased Protein Ubiquitination Levels

**FIGURE 4:**
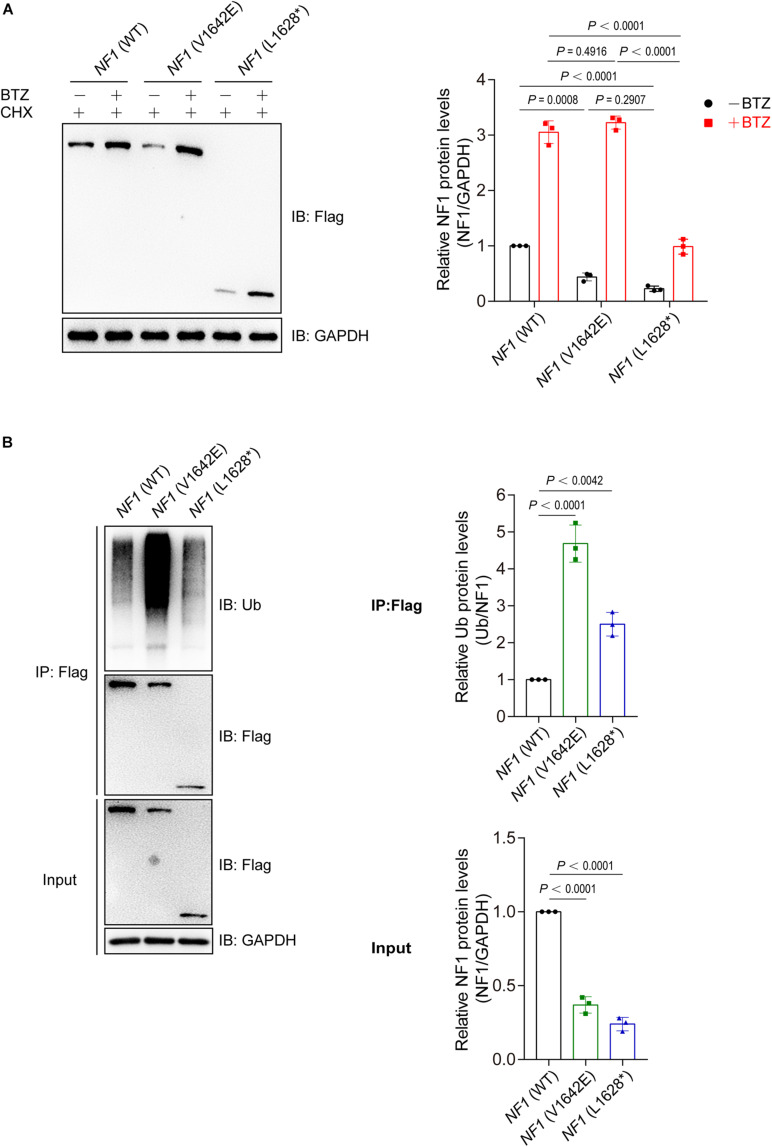
Mutations of *NF1* cause decreased protein stability and increased protein ubiquitination levels. **(A)** Mutants were less stable than wild type NF1 protein. Flag-tagged wild type or mutant NF1 was expressed in SHSY5Y cells, and cells were treated with CHX (100 μg/ml) and with or without BTZ (1 μM) for 6 h before harvest for immunoblotting analysis. BTZ, Bortezomib, a proteasome inhibitor; CHX, cycloheximide, a eukaryote protein synthesis inhibitor. **(B)** Ubiquitination of wild type NF1 protein was less than those of mutants. Flag-tagged wild type or mutant NF1 were expressed in SHSY5Y cells, and immunoprecipitated using anti-Flag beads followed by immunoblotting with anti-Ub to detect ubiquitination signals.

Then we want to explore the reason for phenomenon that showed in Figure 3C. Wild type *NF1* and mutant *NF1* (V1642E and L1628^∗^) were transfected into SHSY5Y cells and treated with or without a proteasome inhibitor Bortezomib (BTZ), as well as cycloheximide (CHX) which is a eukaryote protein synthesis inhibitor. As showed in Figure 4A, BTZ could block the degradation of both wild type and mutant NF1 proteins, but it showed a greater effect on mutants than wild type one, which suggested that mutant NF1 degraded more quickly by proteasome than that of wild type NF1. Further study demonstrated that the ubiquitination of wild type NF1 protein was less than those of mutants, especially mutant NF1 (V1642E) (Figure 4B).

### Mutations of *NF1* Lost the Inhibition on Ras/Erk Signaling and Cell Proliferation

**FIGURE 5:**
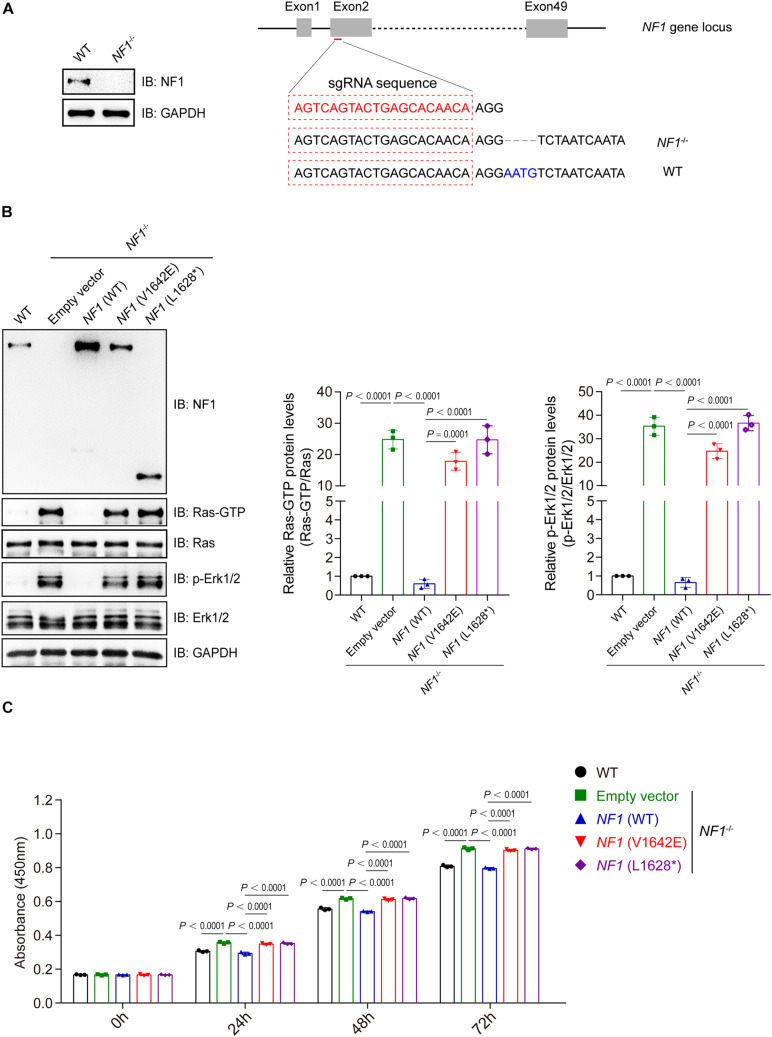
Mutations of *NF1* lost the inhibition on Ras/Erk signaling and cell proliferation. **(A)** A *NF1*^–/–^ SHSY5Y cell line was established using the CRISPR-Cas9 technique. SHSY5Y cells were transfected with CRISPR-Cas9-based sgRNA indicated in red (right panel), monoclonal cells were picked and detected by immunoblotting analysis (left panel), and then genetic ablation of NF1 with a 4-bp deletion was confirmed by sequencing. **(B)** Mutations of *NF1* lost the inhibition on Ras/Erk signaling. NF1^–/–^ SHSY5Y cells were transfected with empty vector, Flag tagged wild type or mutant NF1, and the protein levels of indicated proteins were detected by immunoblotting analysis. **(C)** Mutations of *NF1* lost the inhibition on cell proliferation. The cell viability of wild type SHSY5Y cells, or NF1^–/–^ cells that transfected with empty vector, Flag tagged wild type or mutant NF1, were detected by CCK-8 assay at the indicated time points (0, 24, 48, and 72 h). The 0 h time point was defined as 6 h after the cells were seeded. Data are expressed as mean ± SD and were analyzed using one-way ANOVA with Tukey’s post hoc test, three independent experiments.

A *NF1*-knockout (*NF*^–^*^/^*^–^) SHSY5Y cell line was generated using the CRISPR-CAS9 sgRNA-based method with 4 bp deletion, and was validated by immunoblotting analysis (Figure 5A). *NF1* knockout significantly activates the Ras-GTP and phospho-Erk1/2 signaling in SHSY5Y cells, while re-introduced the wild type but not mutant *NF1* (V1642E and L1628^∗^) could reverse this phenomenon (Figure 5B). The cell viability of wild type SHSY5Y cells was significantly higher than that of *NF1*^–^*^/^*^–^ cells, and re-introduced the wild type *NF1* but not mutant *NF1* in *NF1*^–^*^/^*^–^ cells could restore the inhibition on cell proliferation (Figure 5C).

## Discussion

According to diagnostic criteria by NIH (No author list, 1988a), the signs of multiple CALMs and axillary freckles in our patients are enough to clinically indicate the diagnosis of NF1. In addition, each patient has their respective complaints-bone lesion in patient 1 and recurrent hypertension accompanied with positive family history in patient 2. Routine examination also revealed a short stature and growth hormone deficiency in both patients. It was reported that phenotypic variability is observed in most NF1 patients, even in affected intra- and interfamily. So far, possible explanations include allelic heterogeneity, phenotypes emerging as age progresses, the timing of second hit mutations in different tissues and cells, potential mosaicism in affected patients, modifying genes and environmental factors (Theos and Korf, 2006; Rojnueangnit et al., 2015). Although two mutations described here are not located in the critical GAP-related domain, functional experiments in this study indicated that both mutants had a damaged protein stability and had lost the ability to inhibit Ras/Erk signaling and tumor cell proliferation, which could provide rational interpretations of our patients’ phenotype to some extent.

Previous studies demonstrated that neurofibromin regulates the function of the hypothalamic-pituitary axis in a Ras-independent fashion and that Nf1 deficiency in brain results in decreased GH and IGF levels (Hegedus et al., 2008). Interestingly, two patients in this study were both diagnosed with growth hormone deficiency, which might be mostly responsible for their short stature phenotype. Moreover, functional experiments in this study pointed that the two mutants from our patients can active the Ras/Erk signaling *in vitro*, a proven cascade that has negative relationship with the pathogenesis of bone abnormity in NF1 patients (Wang et al., 2011; Sharma et al., 2013). Collectively, we considered that the growth restriction in our patients was attributed to mutant neurofibromin protein by Ras-dependent and Ras-independent pathways.

Patient 2 in this study presents with hypertension and palpitation. However, clear etiology of hypertension cannot be concluded here. Detailed laboratory and imaging examination excluded the possibility of vascular hypertension, adrenal disease, endocrine system disease, Angio cardiopathy, and pheochromocytoma. It was reported that vascular lesions, predominantly in renal artery, are common causes of secondary hypertension in NF1 patients (Lie, 1998). Smooth muscle or Schwann cells were suggestive of the characteristic nodular aggregates of cells in the abnormal arterial wall in NF1 individuals (Greene et al., 1974; Salyer and Salyer, 1974). Since the CT angiography of the abdominal vessels could not detect small vessels and micrangium, we infer that small vessels abnormality was responsible for the hypertension in patient 2. Considering the progressive nature of this disorder and the tumor proliferation effect observed in our experiment, we suggest a regular check on vessels, especially main artery, should be carried out in case obvious vascular abnormalities would develop later.

In summary, the present study described two NF1 children and tried to provide empirical evidence to interpret related phenotypes. We concluded that dysfunctional neurofibromin protein can account for the short stature phenotype by Ras-dependent and Ras-independent fashion. Patients with hypertension may have negative vascular imaging whereas careful follow-up is necessary in the future. We also observed these two NF1 mutants displayed decreased protein stability with increased ubiquitination levels compared with that of wild-type NF1.

## Data Availability Statement

All datasets generated and analyzed for this study are included in the article/Supplementary Material.

## Ethics Statement

The studies involving human participants were reviewed and approved by Institutional Review Board of the Ruijin Hospital. Written informed consent to participate in this study was provided by the participants’ legal guardian/next of kin. Written informed consent was obtained from the individual(s), and minor(s)’ legal guardian/next of kin, for the publication of any potentially identifiable images or data included in this article.

## Author Contributions

WL and CL: conceptualization, writing—reviewing and editing, and funding acquisition. TZ and TH: methodology and formal analysing. ZD: validation. WL: investigation and supervision. WL and ZD: resources. TZ: data curation and writing—original draft preparation. CL: visualization. All authors have read and agreed to the published version of the manuscript.

## Conflict of Interest

The authors declare that the research was conducted in the absence of any commercial or financial relationships that could be construed as a potential conflict of interest.

## Footnotes

1 http://crispr.mit.edu/
