# Supplementary figures and images for: Characterization of Two Loss-of-Function NF1 Variants in Chinese Patients and Potential Molecular Interpretations of Phenotypes
Source: Front Genet. 2021 May 11;12:660592. doi: 10.3389/fgene.2021.660592 (PMC8144720; doi:10.3389/fgene.2021.660592)

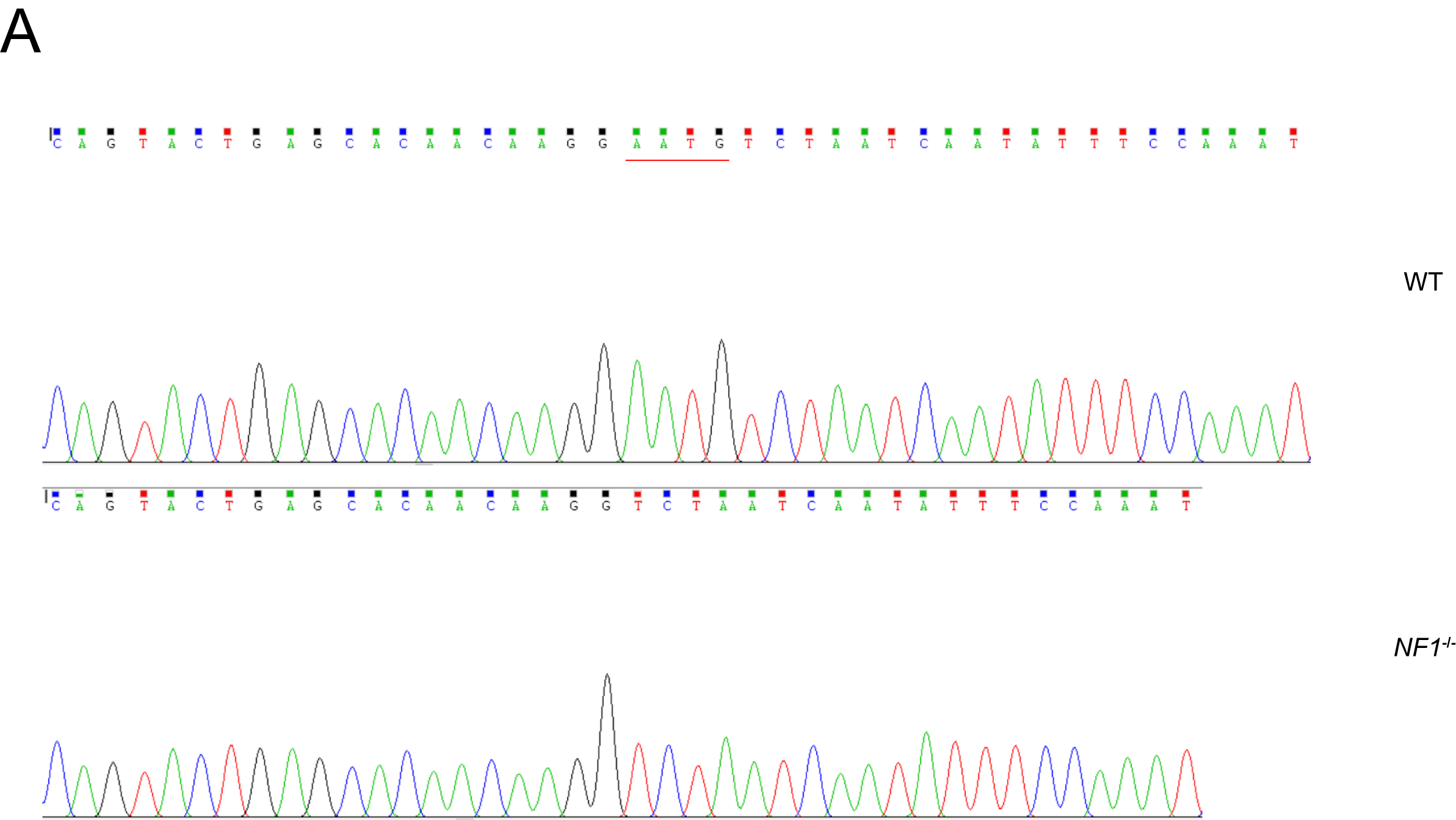

Supplement: Supplementary file 1 [file Image_1.TIF]
